# Bio-Inspired Wooden Actuators for Large Scale Applications

**DOI:** 10.1371/journal.pone.0120718

**Published:** 2015-04-02

**Authors:** Markus Rüggeberg, Ingo Burgert

**Affiliations:** 1 Institute for Building Materials, Swiss Federal Institute of Technology Zürich (ETH Zürich), Zürich, Switzerland; 2 Applied Wood Materials, Swiss Federal Laoratories of Materials Science and Technology (EMPA), Dübendorf, Switzerland; Universita' degli Studi del Salento, ITALY

## Abstract

Implementing programmable actuation into materials and structures is a major topic in the field of smart materials. In particular the bilayer principle has been employed to develop actuators that respond to various kinds of stimuli. A multitude of small scale applications down to micrometer size have been developed, but up-scaling remains challenging due to either limitations in mechanical stiffness of the material or in the manufacturing processes. Here, we demonstrate the actuation of wooden bilayers in response to changes in relative humidity, making use of the high material stiffness and a good machinability to reach large scale actuation and application. Amplitude and response time of the actuation were measured and can be predicted and controlled by adapting the geometry and the constitution of the bilayers. Field tests in full weathering conditions revealed long-term stability of the actuation. The potential of the concept is shown by a first demonstrator. With the sensor and actuator intrinsically incorporated in the wooden bilayers, the daily change in relative humidity is exploited for an autonomous and solar powered movement of a tracker for solar modules.

## Introduction

The design of advanced functional materials with implemented stimuli-responsiveness, programmable and reversible actuation, or shape-memory effect [1–3] is one of the most rapidly developing fields in materials science. Responsiveness is intrinsically incorporated in a variety of materials and external stimuli such as temperature (thermal expansion), relative humidity (swelling and shrinking) or electrical field (piezo effect) [4] can result in reversible dimensional changes. Stimuli-responsive materials have been first successfully synthesized at small scale [4], and a transfer and up-scaling of these dimensional changes into macroscopic programmable movements remains a key challenge for generating adaptive and actuated structures and elements at large scale.

One solution to transform dimensional changes into bending is the bilayer principle. The theory of bending of bilayers has been developed by Timoshenko [5] for bimetal strips exposed to temperature changes. However, actuation of bilayers have also been demonstrated with materials that are responsive to other external stimuli such as relative humidity [6,7], pH [8,9], light [10], or electric fields [11]. By introducing anisotropy in the response of the single layers, even more complex movements can be generated. Depending on the differential orientation of the material in the individual layers, twisting and combinations of twisting and bending of the bilayers can be achieved [6,12].

In nature, several plant species have developed organs which show a bi-layered structure and are reversibly actuated by changes in relative humidity. The scales of pine cones [13] and the awns of the wild wheat [14] reveal bilayer structures which exploit the hygroscopic nature and the pronounced swelling anisotropy of plant tissues. Swelling and shrinking occurs mainly perpendicular to the stiff cellulose microfibrils upon water uptake and loss, whereas the microfibrils prevent largely any swelling parallel to their orientation [15]. By a combination of two layers with different orientations of the cellulose microfibrils bending is generated upon ambient changes of relative humidity. Importantly, in view of the challenges mentioned beforehand, the hygroscopic response at the cell wall level is directly up-scaled to a macroscopic deformation due to the hierarchical structure of the plant tissue. In wood, which largely exhibits an orientation of the microfibrils almost parallel to the long axis of the fibre cells, this leads to the well-known pronounced swelling anisotropy with hardly any swelling and shrinkage occurring in longitudinal direction, but pronounced swelling in radial and especially in tangential direction [15].

In nature, the hygroscopic properties of wood are not utilized for actuation, since the tree keeps the wood in fully hydrated conditions, whereas the swelling and shrinking of timber upon humidity changes is regarded as one of the major drawbacks for its use in construction. However, due to its hierarchical structure and hygroscopic nature wood can also serve as a highly valuable smart material for generating reversible and complex macroscopic movements [16]. Its intrinsic stimuli responsiveness, anisotropy and high stiffness allows for the fabrication of bilayers with differential orientation of the fibres which can be reversibly actuated upon changes of relative humidity. By using wood, the fabrication of anisotropic bilayers is greatly simplified and up-scaling is not limited by the manufacturing process or the lack of mechanically self-supporting capabilities.

Here we demonstrate the general capacity of wooden bilayers to generate controlled macroscopic bending. We use beech and spruce wood for the bilayers, which are common and economically important wood species. Beech wood is in particular suitable for the active layer, as it has a very high swelling coefficient. Amplitude, velocity, and reversibility of the movements were analysed in step-wise intervals and cyclic conditions. The experimental results were compared with the curvatures calculated using the theory of Timoshenko [5], since it was demonstrated that the curvature of anisotropic polymer bilayers actuated by changes in relative humidity can be predicted with this theory [6]. Based on the fundamental understanding of these processes, such wooden bilayers can be integrated in adaptive architectural and constructional elements [16]. As a proof of principle, we introduce a demonstrator in terms of a wooden carrier for solar panels which tracks the sun in the course of the day due to its actuation by the ambient humidity changes.

## Experimental Section

### Material

**Fig 1:**
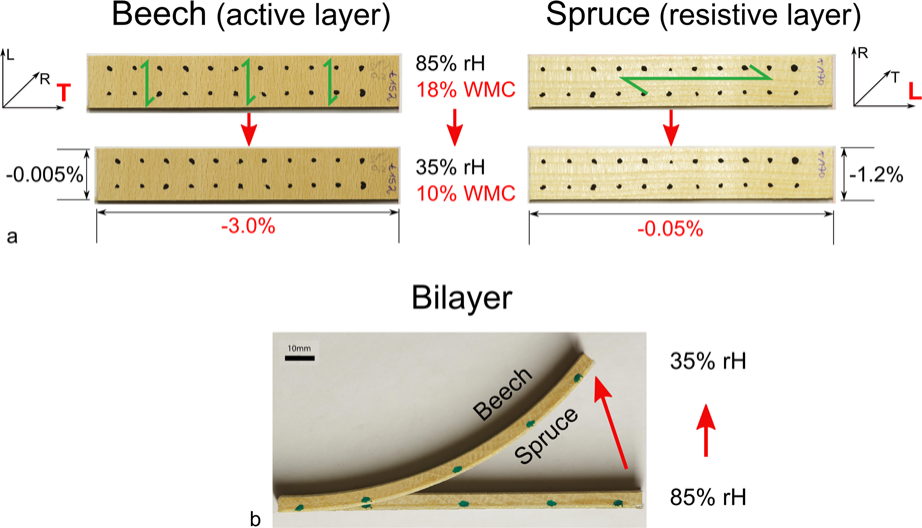
Configuration of beech strips, spruce strips and bilayers and their response to drying. a) Cutting direction of single layers of beech and spruce and their dimensional changes after a decrease in wood moisture content of 10%. L = longitudinal direction, R = radial direction, T = tangential direction; WMC = wood moisture content; rH = relative humidity; green arrows indicate fibre direction and cellulose microfibril orientation (as the microfibrils are oriented almost parallel to the fibre axis). The relative dimensional changes along the long axis of the strips are given in red (specific values for the strips shown). b) Bending of a corresponding bilayer following the change in wood moisture content.

Strips of 120mm length, 20mm width and various thicknesses between 1-4mm were cut from boards of spruce and beech which were obtained from a local wood supplier (Paul Aecherli AG, Regensdorf, Switzerland). Spruce strips with a thickness of 0.2mm and 0.8mm were cut from commercially available sliced veneers (Hess & Co. AG, Döttingen, Switzerland). The beech strips were used as active layer with swelling and shrinking along the long axis of the strip. They were cut with the fibre direction perpendicular to the long axis of the strips with an inclination of the annual rings of 20–25°. This is close to the tangential orientation (Fig. 1a) which is subjected to the largest dimensional changes [15]. The spruce strips were used as resistive layer and cut with the fibre direction parallel to the long axis of the samples and with an inclination of the annual rings of 90° (longitudinal–radial orientation). The strips were stored at 65% relative humidity and 20°C. Prior to gluing and to any experiment the strips and bilayers were equilibrated at the required initial experimental condition for at least 72 hours. Bilayers were prepared (Fig. 1b) by gluing together a beech and a spruce strip with the polyurethane glue HB-S309 (Purbond, Switzerland). The strips and bilayers were weighed immediately before and after gluing and curing to determine the weight of the added glue.

### Step-wise change of relative humidity

Bilayers with a beech layer of 4mm thickness and with a spruce layer of 0.2, 0.8, 1, 2, 3, and 4mm thickness were prepared. For each configuration three bilayers were taken. The spruce and beech strips had been equilibrated and glued in a climatised room operated at 85% relative humidity and 20°C. Green marker dots were applied on one narrow side of each bilayer (Fig. 1b). In addition, two sets of six beech strips (thickness of 4mm) and four spruce strips (thickness of 1, 2, 3, and 4mm) were included in the experiment. One set of strips was coated with aluminium foil on one side to estimate wood moisture change of the beech and spruce layer separately. The broad sides of the strips of the second set were marked with black dots (Fig. 1a) to determine the differential swelling coefficient (see below). The bilayers and strips were transferred to a different climatised room operated at 35% relative humidity and 20°C. The weight was recorded and images were taken at 0, 1, 2, 4, 6, 8, and 24 hours after the transfer.

### Cyclic change of relative humidity

Curvature and weight were also recorded in cyclic conditions. The two parameters could not be recorded simultaneously for the same sample. For measuring curvature, four bilayers of the static experiments were taken with the spruce layer thickness being 0.2, 0.8, 1, and 2mm. For weight recordings five beech strips were taken, which were covered with sticky aluminium foil on one side. The bilayers were placed into a programmable climatic chamber. Relative humidity was varied at constant rates between 85% and 35% with cycles of 24, 12, and 6 hours with four loops for each interval. Temperature was kept constant at 20°C. Relative humidity and temperature were recorded with a combined humidity and temperature sensor (HC2-C05 sensor, Rotronic, Switzerland) every minute. The weight was recorded every ten seconds. Images of the bilayers for determining curvature were taken every five minutes through the front window of the climatic chamber with a digital SLR camera and a studio flash light.

### Field experiment

The long-term pattern of the actuation of wooden bilayers in full weathering conditions was tested in a field experiment at ETH Zurich. The experiment started in July 2013 and ran for nine months. Two bilayers were loaded with 100g and 150g respectively. Images of the bilayers were taken every five minutes with a C-mount digital camera equipped with a zoom lens, which was fixed on the table inside a weather protection housing. A weather station next to the table recorded temperature, relative humidity, wind speed and direction, solar radiation, and precipitation every 15 minutes.

Curvature of the bilayers were determined for days without rain at maximum relative humidity (typical in the morning just before sun rise) and minimum relative humidity (typical at late afternoon). Five points along each bilayer were manually recorded. The curvature was then calculated as described below. Down-bending of the sample was hereby defined as negative curvature, up-bending as positive curvature.

### Wood moisture content

The wood moisture content of the samples was determined by oven drying reference samples at 104°C for 24h. Afterwards the samples were stored in vacuum at room temperature for 30 minutes and then immediately weighed. The wood moisture content was then determined using Equation 1 with u = wood moisture content, m_u_ = weight of the sample at the wood moisture content u and m_dr_ = dry weight.

$$u\;=\;\frac{m_{u}-m_{dr}}{m_{dr}}∙100\%$$ (1)

### Differential swelling coefficient

The differential swelling coefficient was determined by tracking the dimensional changes of the marked spruce and beech strips included in the experiment with step-wise change of relative humidity (see above). Tracking was performed by analysing the images of the strips with ImageJ. With ε being the strain, u being the wood moisture content (Equation 2) and u_0_ being the initial wood moisture content, the differential swelling coefficient is

$$\propto \;=\;\frac{\varepsilon }{\left(u-u_{0}\right)}$$ (2)

As relative humidity was changed from 85% to 35%, the coefficients were obtained as shrinking coefficients.

### Experimental determination of curvature

The coordinates of the marker dots were tracked with ImageJ and circles were fitted to the coordinates by using the least-square optimizing function of the Python package Scipy. The curvature was taken as the inverse radius.

### Calculation of curvature using Timoshenko’s theory

Timoshenko [5] has calculated the curvature of bimetal thermostats subjected to a temperature change c-c_0_ (Equation 3). Temperature is substituted by wood moisture content and the specific expansion coefficients α_1_ and α_2_ are taken from measurements of reference samples. The longitudinal stiffness of spruce E_1_ and the tangential stiffness of beech E_2_ were taken from literature [17,18]. With the layer thicknesses h_1_ and h_2_ and the total thickness of h the curvature of the wooden bilayers is calculated by Equation 3. Hereby the factor k is termed as the specific curvature.

$$\frac{1}{\rho }\;=\;\frac{6{\left(1+m\right)}^{2}}{\left(3{\left(1+m\right)}^{2}+\left(1+mn\right)\left(m^{2}+\frac{1}{mn}\right)\right)}\frac{\left(\alpha_{2}-\alpha_{1}\right)\left(c-c_{0}\right)}{h}\;=\;k\frac{\Delta \alpha \Delta c}{h},\;m\;=\;\frac{h_{1}}{h_{2}},\;n\;=\;\frac{E_{1}}{E_{2}}$$ (3)

The force F_c_ induced by the curvature of a bilayer strip of length l is calculated by Equation 4 [5]. Hereby E and I of the bilayer strip are approximated by taking the weighted average values. The curvature for mechanically loaded bilayers can then be calculated by using Equation 4 and replacing F_c_ with the difference of forces F_c_-F_m_, F_m_ being the force induced by the load.

$$\frac{l^{2}}{2\rho }\;=\;\frac{F_{c}l^{3}}{3EI}$$ (4)

The angular change of the bilayer is calculated with Equation 5.

$$\varphi \;=\;\frac{l}{\rho }∙\frac{360}{2\pi }$$ (5)

## Results and Discussion

### Step-wise change of relative humidity

**Fig 2:**
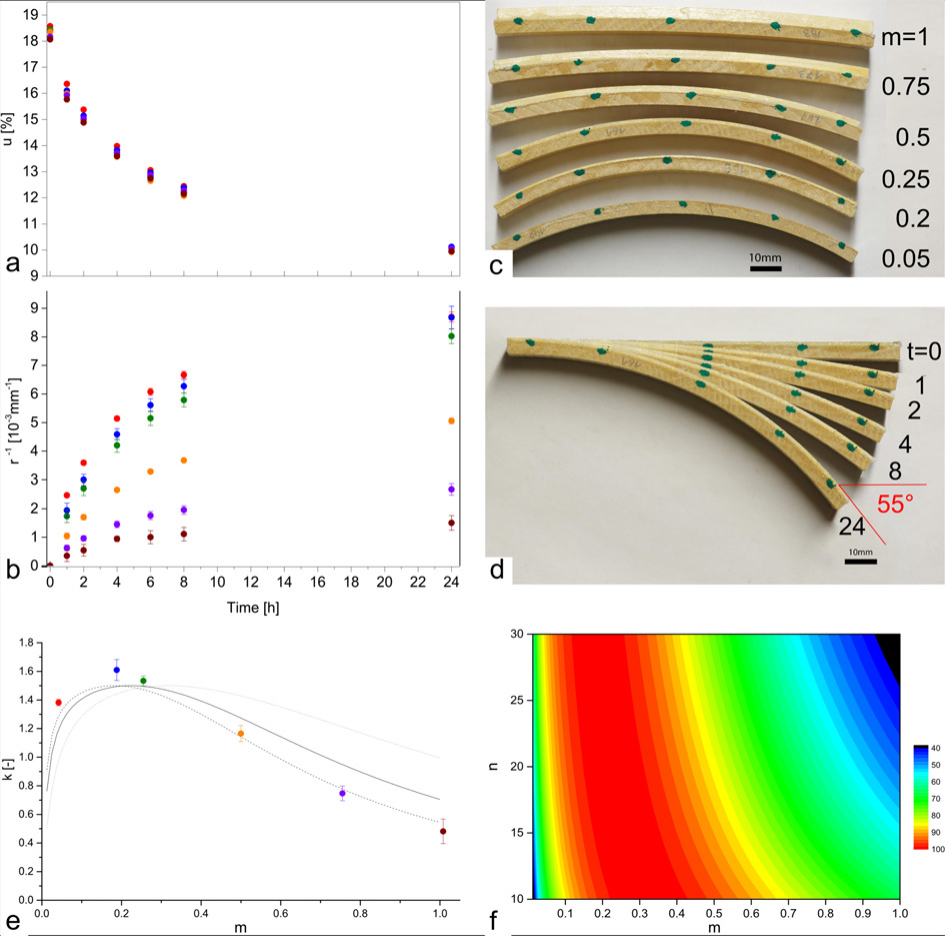
Actuation of bilayers after step-wise change of relative humidity from 85% to 35%. Color code: red: 0.2mm spruce layer (thickness ratio m = 0.05); blue: 0.8mm (0.2), green: 1mm (0.25), orange: 2mm (0.5), purple: 3mm (0.75), brown: 4mm (1). a) Wood moisture content, b) curvature, c) curvature of bilayers with different m (note that the overall thickness h also changes), d) curvature of a bilayer 1,2,4,8, and 24 hours after transfer. The actuation is also made visible in a movie (S1 Movie). e) Comparison of experimentally derived and calculated specific curvature k 24 hours after transfer. Calculations are shown for values of n = E1/E2 of 10 (dotted line), 20 (solid line), and 30 (patched line). f) Relative amplitude of k as a function of n and m.

In analogy to the biological role models (pine cone, wheat awn) wooden bilayers were exposed to a step wise change of relative humidity from 85% to 35%. Samples with different bilayer constitutions were tested with the thickness of the resistive spruce layer being varied in the range of 0.2 to 4mm, whereas in all cases the beech layerwas 4mm thick. After the step-wise change of relative humidity, wood moisture content strives towards a new equilibrium moisture content. A decrease of the wood moisture content from around 18% to around 10% within 24 hours was observed (Fig. 2a) and the initially straight bilayers became increasingly curved (Figs. 1b and 2b). While the amplitude of wood moisture change was very similar for all bilayers, the amplitude of curvature was negatively correlated with the thickness of the spruce layer for thicknesses greater than 1mm. For bilayers with a thickness of the spruce layer up to 1mm, curvatures up to 8.7*10^-3^mm^-1^ were reached, whereas bilayers with a spruce layer of 4mm thickness reached a curvature of 1.5*10^-3^ mm^-1^ after 24 hours only. Fig. 2c presents the curved bilayers with the different ratios of layer thickness after 24 hours. The actuation over time is made visible as composite image in Fig. 2d and in a movie (S1 Movie). With the length of the bilayers being 120mm, the change in curvature of 8.7*10^-3^mm^-1^ results in an angular change of 59° (Equation 4), which is close to the measured change of 55° (Fig. 2d).

The experimentally derived specific curvatures which were reached 24 hours after transfer matches with those which were calculated using the theory of Timoshenko (Fig. 2e), which confirms the results of preceding studies on moisture induced actuation of polymer bilayers [6]. For the calculation, the differential swelling coefficient had to be calculated. Since relative humidity was altered from 85% to 35% the swelling coefficient was obtained as shrinking coefficient. For beech strips, a differential coefficient of 3.2±0.1*10^-3^/% was obtained, for the spruce strips the coefficient was 0.7±1.0*10^-4^/%. Since the spruce layer acts as resistive layer, the curvature solely depends on the wood moisture content of the beech layer. Calculations on the combined wood moisture content of beech and spruce layers reveal that the wood moisture content of the bilayer can be taken as a sufficiently precise approximation of the wood moisture content of the beech layer (S1 Fig.).

Stiffness ratios in the range of 10 to 30 were used in the calculations as literature reports values of 10-15GPa for the longitudinal stiffness of spruce [18] and 0.5–0.8GPa for the tangential stiffness of beech [17,19]. Due to the high stiffness ratio, the amplitude of curvature is strongly dependent on the thickness ratio of the spruce and the beech layer and shows an optimum between 0.2 and 0.3. Thus for the wooden bilayers in the present configuration the amplitude of curvature is mainly dictated by the thickness ratio rather than the stiffness ratio (Fig. 2f).

The specific curvature of the wooden bilayers was also calculated for the non-equilibrium state (S2a Fig.) and matches with the theoretically calculated one. However, the behavior of wooden bilayers is more complex in this state as the swelling coefficient, which was measured with six beech and six spruce strips, is not constant over time, but showed an increase for beech within the 24 hours (S2b and c Figs.).

### Cyclic conditions

**Fig 3:**
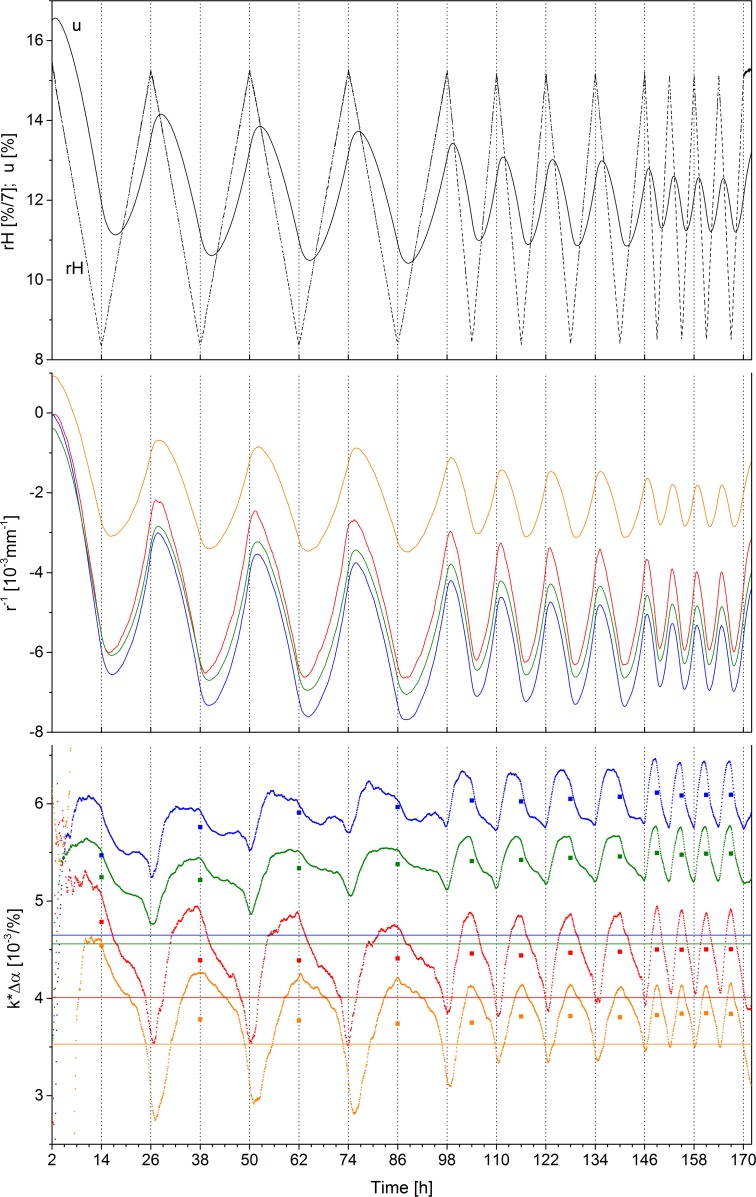
Actuation of bilayers during cyclic change of relative humidity. a) Relative humidity and wood moisture content, b) curvature of bilayers (color code as in Fig. 2). c) Specific curvature k*Δα: experimentally derived (small dots), average of each loop (squares), and calculated using Equation 3 (lines).

Since the water exchange in wood via sorption and diffusion takes time, the new equilibrium state in the step wise changes was reached after approximately 24 hours. In practical applications the bilayers will be subjected to gradual and cyclic changes and, thus, will be operated in non-equilibrium state which results in a more complex behaviour. Hence, the actuation pattern in cyclic conditions was investigated for four bilayers made of beech and spruce with varying spruce layer thicknesses. These bilayers were subjected to a cyclic change of relative humidity between 85% and 35% with cyclic periods of 24, 12, and 6 hours, respectively (Fig. 3a). For each period four loops were programmed. The cyclic change in relative humidity resulted in changes of wood moisture content of 3.3±0.2% for a cycle of 24 hours, 2.1% for 12 hours and 1.3% for 6 hours. For a bilayer with 0.2mm thick spruce layer this change in wood moisture content resulted in amplitudes of curvature of 4.1±0.4*10^-3^mm^-1^, 2.9±0.2*10^-3^mm^-1^, and 2.0±0.05*10^-3^mm^-1^, respectively (Fig. 3a-b). Table 1 gives an overview of the curvatures achieved with the other three bilayers. The amplitudes stayed almost constant during the four loops of one specific cycle. The wood moisture content could not be measured on the bilayers themselves, but was measured on additional beech strips on a scale within the climate chamber.

**Table 1:** Wood moisture content, curvatures and phase lags for bilayers with different thickness ratios in cyclic conditions.

Cycle [h]^a^)	Δu [%]	Δ r^-1^ 0.2/4mm^a^ [10^-3^mm^-1^]	Δ r^-1^ 0.8/4mm [10^-3^mm^-1^]	Δ r^-1^ 1/4mm [10^-3^mm^-1^]	Δ r^-1^ 2/4mm [10^-3^mm^-1^]	Ph.lag u^b^ [h&rad]	Ph.lag r^-1^ ^b^ [h&rad]	Ph.lag u^c^ [rad]	Ph.lag dim.^c^ ^,^ ^d^ [rad]
**24**	3.3(0.2) ^f^	4.1(0.4)	3.8(0.5)	3.6(0.4)	2.5(0.2)	2.5(0.4)^e^	1.6(0.3)		
						0.6(0.1)	0.4(0.1)	0.31	0.29
**12**	2.1(0.0)	2.9(0.2)	2.2(0.2)	2.2(0.2)	1.6(0.2)	1.4(0.1)	1.0(0.1)		
						0.7(0.1)	0.5(0.1)		
**6**	1.3(0.0)	2.0(0.1)	1.5(0.1)	1.5(0.1)	1.0(0.0)	0.8(0.1)	0.6(0.1)		
						0.8(0)	0.7(0.1)	0.44	0.39

^a)^ Thickness of spruce and beech layer;

^b)^ Ph.lag = phase lag, present measurements for all bilayers;

^c)^ taken from Ma et al.[21], dim = dimension;

^d)^ average of radial and tangential response;

^e)^1^st^ row: phase lag in hours, 2^nd^ row: phase lag in radians for comparison with the measurements of Ma et al.;

^f)^ standard deviation in parenthesis.

A phase lag between relative humidity and wood moisture content was observed. Whereas its absolute (mean) value decreased from 2.5h to 0.8h for the successively shorter cycles its relative value (expressed in radians) increased for shorter cycling periods (Table 1). For the curvature a phase lag exists as well, but, remarkably, the phase lag is smaller than that of the wood moisture content (decreasing from 1.6h to 0.6h), which means that the change in curvature precedes any measureable change in wood moisture content. These phase lags result in a pronounced hysteresis between curvature and wood moisture changes (S2 Fig.). This effect was also reported by Chomcharn & Skaar and by Ma et al. [20,21] but to a lesser extent. In their study, dimensional changes of wood samples preceded changes in wood moisture content in cyclic conditions. This effect was explained by changing gradients of wood moisture content within the sample resulting in a lower phase lag for the dimensional change compared to that for wood moisture content.

Specific curvature was derived from the experiment in terms of k*Δα (Fig. 3c), as the swelling coefficient could not be measured at the same time. The specific curvature showed considerable changes within one loop, which may be one effect of the hysteresis. However, the average specific curvature of one cycle remains nearly constant across the four loops for one specific cycle period and across the different cyclic periods. For the bilayers with 0.2mm and 0.8mm thick spruce layers, the specific curvatures slightly increased over time and were 11% and 13% higher than the calculated values. These discrepancies may be explained by the fact that the swelling coefficients could not be measured during this experiment and the wood moisture content was measured on different samples. For the other two bilayers differences between experimentally derived and calculated specific curvature were less than 5%.

### Long-term actuation under full weathering

**Fig 4:**
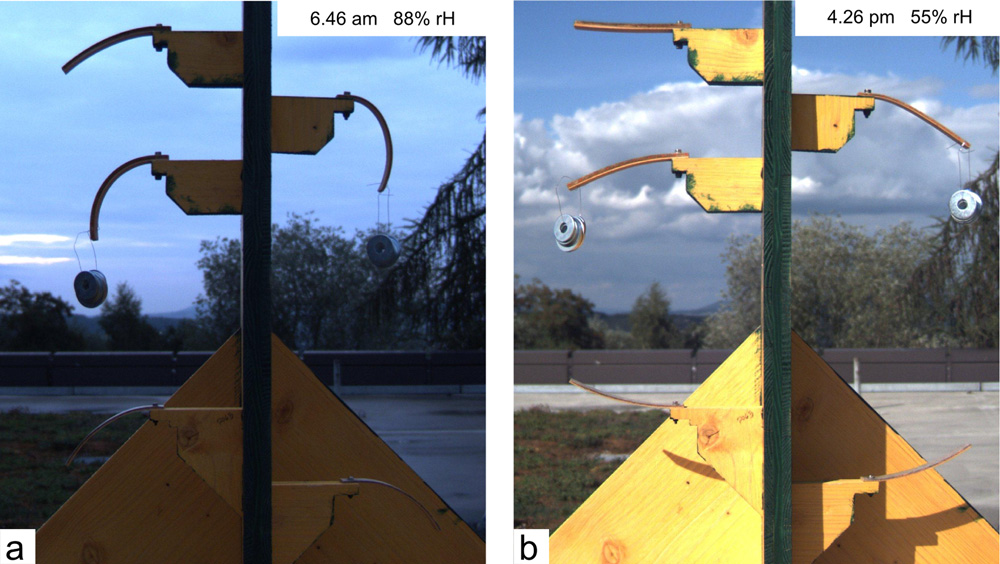
Wooden bilayers in the long-term field experiment. a) Down-bending of the bilayers in the morning on Sept. 13th 2013, b) Up-bending of the bilayers in the afternoon. Two bilayers are mechanically loaded. The actuation of the bilayers on that day is shown in the S2 Movie.

**Fig 5:**
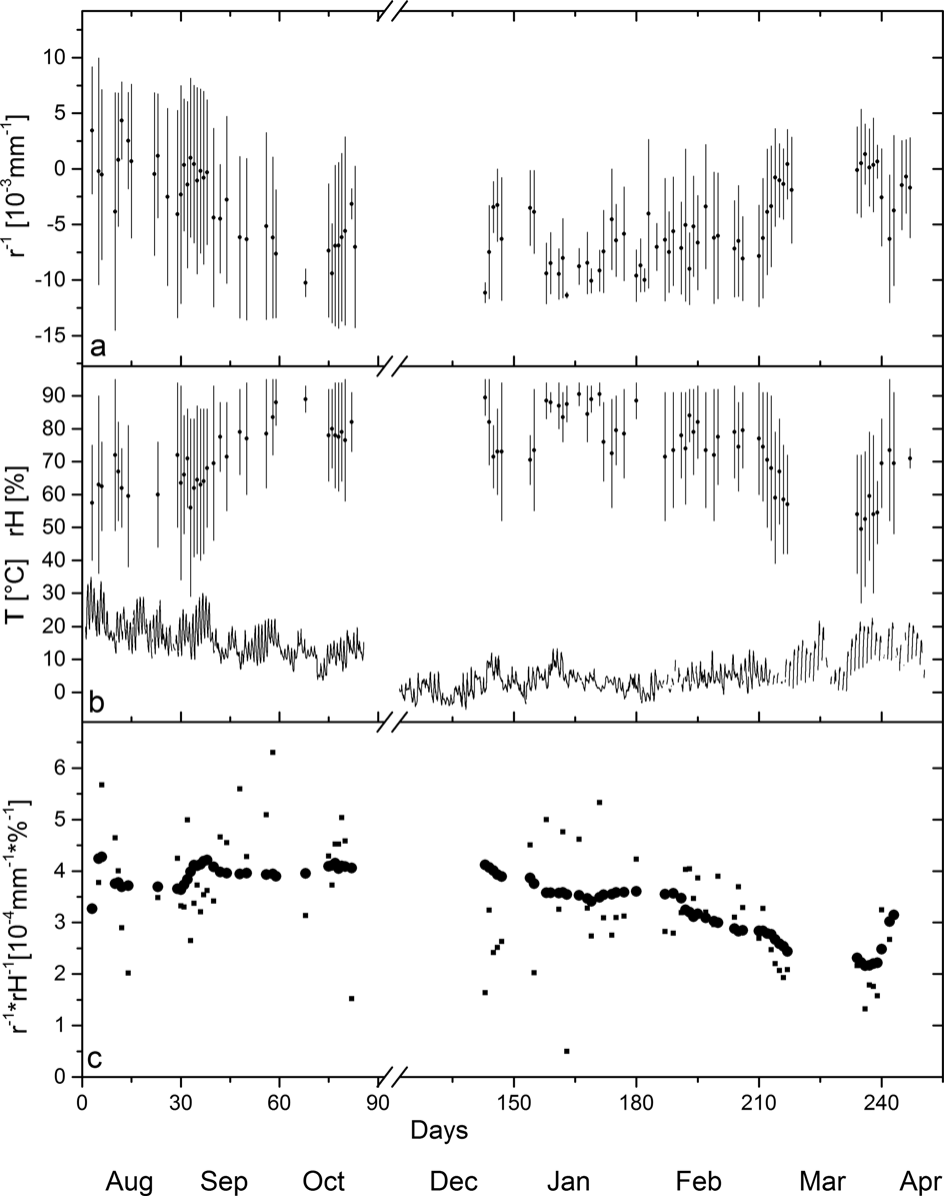
Long-term actuation of bilayers during field test in full weathering conditions. a) Daily change of curvature (lines), and mean (sliding average over 11days) curvature (dots), b) daily change of relative humidity (lines), mean relative humidity (dots), and temperature, c) ratio of curvature change and change in relative humidity (small squares) and average ratio (sliding average with a window of 11 data points) (dots).

Besides amplitude and response time of the actuation, the durability of the actuators, and the stability of the actuation over time are crucial factors for practical application. In a long-term field test wooden bilayers were exposed to full weathering conditions (Fig. 4). To maintain the maximum possible water exchange and amplitude of curvature, the bilayers were kept untreated and unprotected. With the beech layer facing upwards, the bilayers reached their maximum down-bending position (negative curvature) in the early morning when maximum relative humidity was observed (Fig. 4a). During daytime the bilayers gradually bent upwards and reached their maximum up-bending position (positive curvature) in the late afternoon when the minimum of relative humidity was reached (Fig. 4b & S2 Movie). Two bilayers were mechanically loaded. The effect of mechanically loading will be discussed below. Fig. 5a shows exemplarily the amplitude of curvature of one bilayer with a 2.5mm thick beech layer and a 1mm thick spruce layer over a period of nine months. Curvature was evaluated for days without rain and a minimum change of 5% in relative humidity. The daily amplitude of curvature was in the range of 7±3.7*10^-3^ to 10±4.5*10^-3^mm^-1^ for bilayers with a 2.5mm thick beech layer and a 1mm thick spruce layer whereas the bilayers with a 4mm thick beech layer and a 1mm thick spruce layer showed amplitudes in the range of 2.4±1.5*10^-3^ to 4±2.5*10^-3^mm^-1^. Thus angular changes in the range of 48±25° to 69±31° were achieved for the thin bilayers whereas the thick bilayers showed angular changes in the range of 17±10° to 28±17°.

Data of relative humidity and temperature are presented in Fig. 5b. The daily change in relative humidity is highly variable. For the days with a change in relative humidity of more than 5%, an average daily change in relative humidity of 30±12% was calculated. Apart from the high daily variation in the amplitude of relative humidity, seasonal variation occurred. Within the first three months throughout late summer and autumn, a gradual increase of the mean relative humidity and a gradual decrease of the mean temperature (Fig. 5b) was observed. These changes in climatic conditions result in a higher wood moisture content and thus affect the curvature of the bilayers which gradually bent more downwards. From the end of February onwards the trend from autumn was reversed with a decreasing mean relative humidity, increasing temperature and in consequence an up-bending of the bilayers.

To obtain information on the long-term stability of the amplitude of actuation, curvature was correlated to the course of relative humidity changes (Fig. 5c). On direct comparison the measured curvature and relative humidity showed only weak correlation, which is most probably caused by additional influencing factors such as temperature, solar radiation, wind, or the preceding rate of change of relative humidity. However, by averaging over time (sliding average over 11days), a trend in this ratio could be revealed. From August 2013 until end of January 2014 the average specific curvature remained within the range of 3–4*10^-4^mm^-1^%^-1^. From February to April 2014 it decreased to 2-3*10^-4^mm^-1^%^-1^ and then slightly increased again (Fig. 5c). Hence, although splitting and cracking of the wood due to the impact of UV-radiation and rain became visible, the amplitude of actuation has only slightly decreased over time.

### Adapting the amplitude of actuation

The amplitude of actuation and the response time are crucial factors in practical application defining limits of operation and sensitivity. Using thin bilayers leads to a higher amplitude of curvatures compared to thick bilayers as the amplitude of curvature proportionally increases in case of a decrease of the thickness (Equation 3). Curvature changes also occur in shorter times for thin layers (S2 Fig.) which leads to a further increase in the amplitude of curvature in cyclic condition. Stability requirements and mechanical impact may impede the use of thin bilayers, but the amplitude of actuation can be further varied by adapting the length of the bilayer, since the angular change of the free end of the bilayer is directly proportional to the length of the bilayer for a given curvature change (Equation 5).

### Mechanical loading

**Fig 6:**
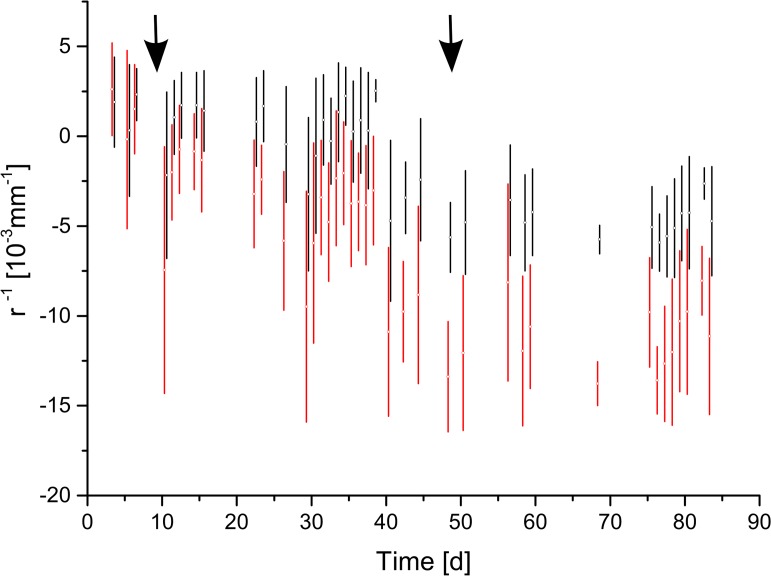
Mechanical loading of a bilayer in the field experiment. Colour code: black: unloaded bilayer (3.8mm beech, 2mm spruce), red: bilayer (4mm beech, 1.6mm spruce) loaded with a point load of 100g at the free end of the bilayer. Arrows indicate application and removal of the weight.

Mechanical load may cause creep in the viscoelastic wood elements with severe impact on the actuation capability. Hence, the effect of mechanical loading on the actuation was analyzed for two bilayers within the field experiment for three months (Fig. 4 & S2 Movie). These bilayers were loaded with point loads of 100g and 150g respectively at their free ends. The bilayers gradually bent more downwards compared to the unloaded bilayers (Fig. 6). This altered equilibrium position remained after removing the weight indicating that creep with plastic deformation has been induced by this permanent mechanical loading. However, more importantly hardly any decrease occurred in the amplitude of actuation. Calculations using Equation 4 confirm that curvature is reduced by less than 3% by loads of 1–1.5N for the wooden bilayers used in this experiment.

### Solar tracker for solar panels

**Fig 7:**
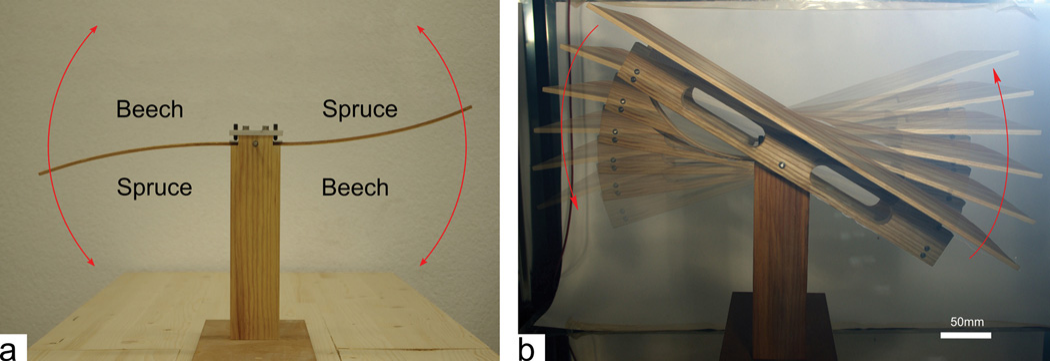
Prototype of a solar tracking carrier for solar modules. a) Set-up showing the weight carrying column with the two bilayers attached in reversed configuration, red arrows indicate reversible actuation when relative humidity is altered. b) Composite image showing the resulting rotation of the attached platform within three hours after change from 35% to 85% relative humidity. The rotation of the platform is also shown in a movie (S3 Movie).

Based on the extracted principles of moisture induced deformation, a demonstrator of a bio-inspired solar tracking carrier for solar modules was constructed and tested in the climate chamber (Fig. 7). Two wooden bilayers with inversed configuration were used as “motor” (Fig. 7a). One end of the bilayers is fixed at the load carrying central column. The other end is connected to the rotating platform (Fig. 7b), hereby allowing the sliding of the bilayers at the connection during rotation. By changing relative humidity from 35% to 85% a rotation of the platform of 50° was achieved within three hours (Fig. 7b, S3 Movie). This setup resembles a tee-totter, and a maximum rotation of the platform of 90° is achievable for an angular change of the bilayers of 180°.

The solar tracking carrier for solar modules demonstrates the feasibility of using bilayers for autonomously controlled outdoor applications. Ultimately, humidity changes drive the tracking of the sun by actuating the bilayers, which allows to direct the solar module towards the east in the morning, let it turn to the west in the course of the day upon decreasing relative humidity and let it retract at night at high relative humidity.

In the setup of a solar tracker the wooden bilayers are used to power the rotation of the rigid platform rather than representing motor and actuated element in one part. This concept enlarges the design space of convertible elements and the field of possible applications as the element to be moved can be independently designed from the wooden bilayer. In case of the solar tracker the wooden bilayer are protected by the solar panel and are not directly exposed to sunshine and rain, which will increase their lifetime and decrease the daily variability of actuation. However, mechanical loading should be minimized or better avoided as creep involving plastic deformation will occur in the bilayer even in low level loading conditions, as the field test has shown for loadings of 1–1.5 N (Fig. 6). For the solar tracker the principle of the tee-totter minimizes mechanical loading of the bilayers, since the weight of the rotating platform is carried by the static central column whereas the actuators only have to overcome the rotation inertia of the platform.

When using convertible wooden elements for actuation, the precision of regulation is limited mainly by the intrinsic variability and the response time. For those applications, which tolerate such a variability in the deformation pattern, the presented actuation by wooden bilayers provides a system which is strikingly simple and cheap to produce and shows long-term stability despite the impact of weathering.

## Conclusion

The hygroscopic nature of wood and its dimensional instability in response to water uptake and loss was utilized to develop humidity driven actuators employing the bilayer principle. The actuation pattern is in accordance with the theory of bending of bilayers and can thus be predicted. Amplitude and response time can be adapted by the geometry of the bilayer. Wooden bilayers are especially suitable to power outdoor convertible elements, as the daily, solar driven alteration in relative humidity is taken as energy source and actuation persists despite the impact of weathering. The requirements for actuation do not have to be imprinted via a specified and complex manufacturing process but are intrinsically incorporated in the material itself. The strikingly simple manufacturing process and the excellent mechanical properties of wood facilitate the up-scaling of the actuators. Thus, wood offers a unique combination of physical, structural and mechanical properties for its use as actuator in large scale applications. As wood is a natural, biodegradable material, actuation by wooden bilayers represents a sustainable way to power the movement of architectural and constructional elements.

## Supporting Information

S1 Fig Wood moisture content u over time of single wood layers after transfer from 85% to 35% relative humidity. Moisture content of a single beech layer (open circles, 4mm thick), spruce layers of different thickness (open squares: 1mm, open triangle up: 2mm, open triangle down: 3mm, open triangle left: 4mm) and calculated combined wood moisture content of beech and spruce layer (closed circles) after transfer from 85% to 35% relative humidity. The wood moisture content of the beech layer within a bilayer can be approximated by the wood moisture content of the entire bilayer.
(TIF)

S2 Fig Specific curvature k and swelling coefficients α (taken as shrinking coefficients) after transfer from 85% to 35% relative humidity. Colour code for (a) and (c) as in Fig. 2. a) Specific curvature k over time, points: experimentally derived values, lines: values calculated using the theory of Timoshenko (Equation 3). b) Shrinking coefficients α (ε/Δu) of beech (open circles) and spruce (open triangles) and the difference of coefficients Δα (closed squares) over time. c) Experimentally derived specific curvature k*Δα.
(TIF)

S3 Fig Curvature of two bilayers as a function of wood moisture content u in cyclic condition. Cycles of 24h, 12h, and 6h with four loops for each cycle are shown. Thickness of the beech layer: 4mm, thickness of the spruce layer: 0.2mm (red) and 2mm (orange).
(TIF)

S1 Movie Actuation of a bilayer (4mm beech/1mm spruce layer) after transfer from 85% to 35% relative humidity. The actuation shown in this movie is represented by the composite image in Fig. 2d. Length: 3s, real time: 15.25h.
(AVI)

S2 Movie Actuation of bilayers in full weathering condition on Sept. 13^th^ 2013. Note that two bilayers are mechanically loaded. The starting time of the movie is 4.45am. The actuation is represented by the two images of Fig. 4 showing maximum down- and up-bending in the morning and in the afternoon respectively. Length: 9s; real time: 19.5h.
(AVI)

S3 Movie Rotation of the solar tracking platform for solar panels after transfer from 35% to 85% relative humidity. The movie is represented by the composite image in Fig. 7b. Length: 5s, real time: 3h.
(AVI)
