# Supplementary figures and images for: Bio-Inspired Wooden Actuators for Large Scale Applications
Source: PLoS One. 2015 Apr 2;10(4):e0120718. doi: 10.1371/journal.pone.0120718 (PMC4383548; doi:10.1371/journal.pone.0120718)

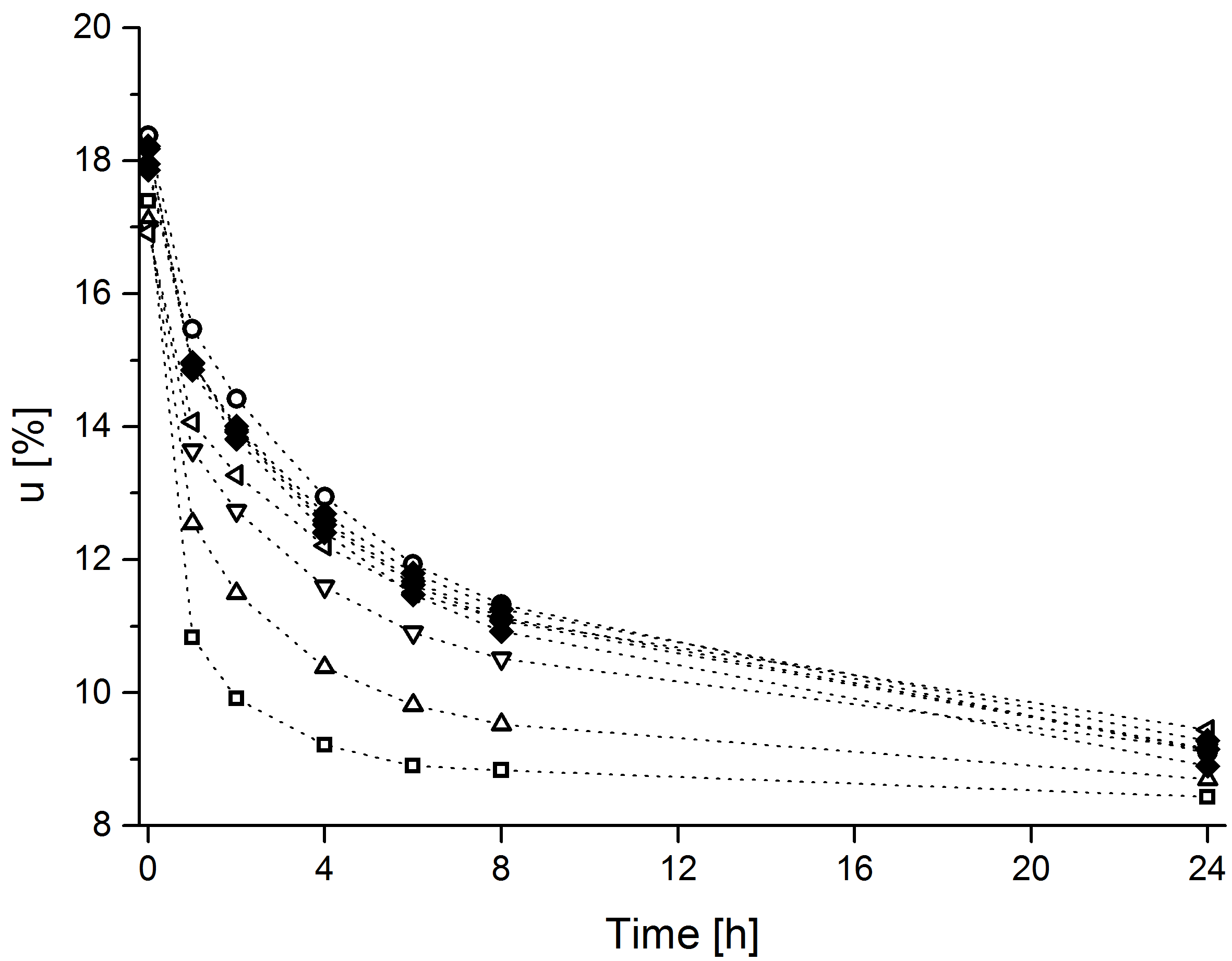

Supplement: S1 Fig — Moisture content of a single beech layer (open circles, 4mm thick), spruce layers of different thickness (open squares: 1mm, open triangle up: 2mm, open triangle down: 3mm, open triangle left: 4mm) and calculated combined wood moisture content of beech and spruce layer (closed circles) after transfer from 85% to 35% relative humidity. The wood moisture content of the beech layer within a bilayer can be approximated by the wood moisture content of the entire bilayer. (TIF) [file pone.0120718.s001.tif]

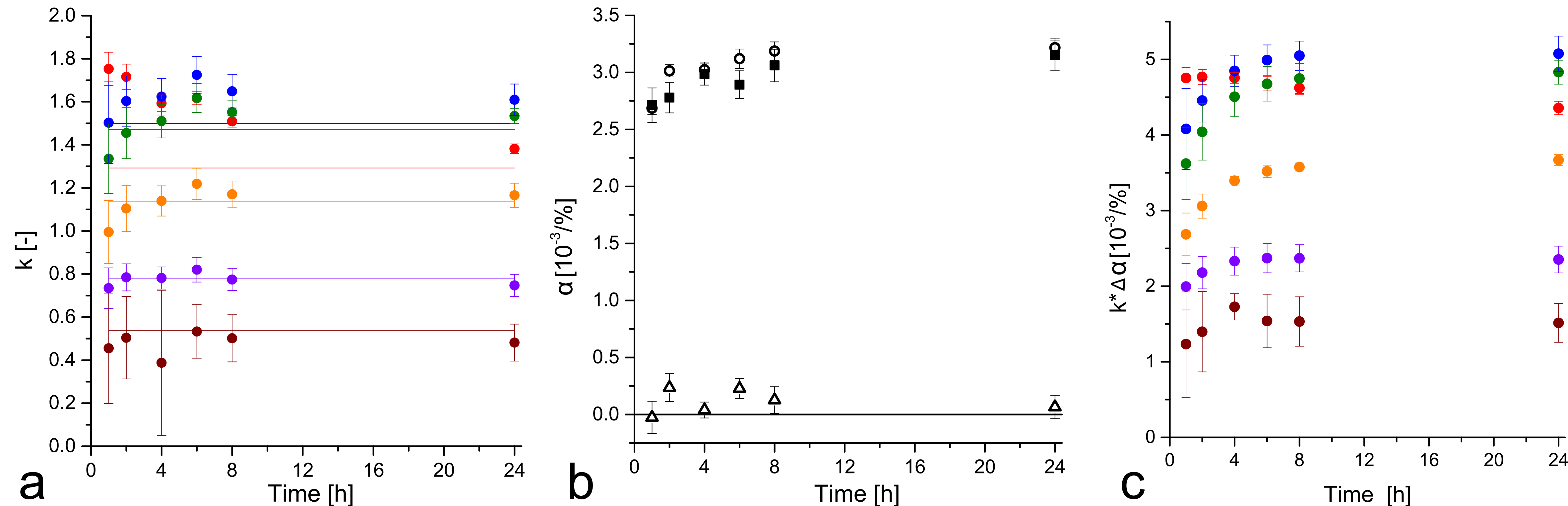

Supplement: S2 Fig — Colour code for (a) and (c) as in Fig. 2. a) Specific curvature k over time, points: experimentally derived values, lines: values calculated using the theory of Timoshenko (Equation 3). b) Shrinking coefficients α (ε/Δu) of beech (open circles) and spruce (open triangles) and the difference of coefficients Δα (closed squares) over time. c) Experimentally derived specific curvature k*Δα. (TIF) [file pone.0120718.s002.tif]

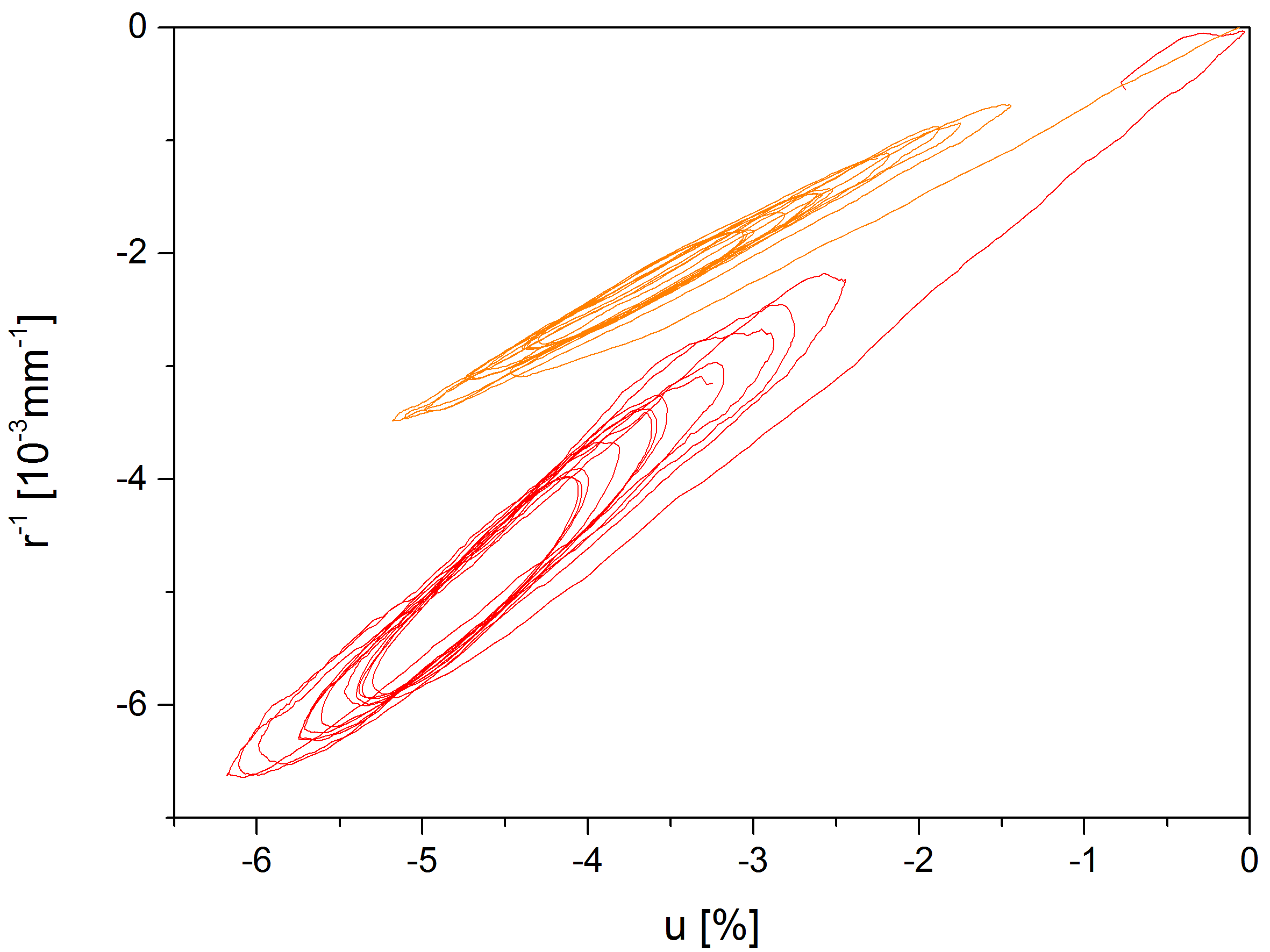

Supplement: S3 Fig — Cycles of 24h, 12h, and 6h with four loops for each cycle are shown. Thickness of the beech layer: 4mm, thickness of the spruce layer: 0.2mm (red) and 2mm (orange). (TIF) [file pone.0120718.s003.tif]
